# Malnutrition–Inflammation Score of Patients with Chronic Kidney Disease from Early Stage to Initiation of Dialysis

**DOI:** 10.3390/nu16234014

**Published:** 2024-11-23

**Authors:** Lee-Moay Lim, Hung-Tien Kuo, Yu-Lin Chao, Feng-Ching Shen, Yi-Kong Chen, Yi-Wen Chiu, Shang-Jyh Hwang, Chi-Chih Hung

**Affiliations:** 1 Division of Nephrology, Department of Internal Medicine, Kaohsiung Medical University Hospital, Kaohsiung Medical University, Kaohsiung 807378, Taiwan; limleemoay@gmail.com (L.-M.L.); 2 School of Medicine, College of Medicine, Kaohsiung Medical University, Kaohsiung 807378, Taiwan

**Keywords:** chronic kidney disease (CKD), malnutrition–inflammation score (MIS), protein energy wasting (PEW), nutritional assessment, dialysis

## Abstract

**Background:** The malnutrition–inflammation score (MIS) is a practical and accessible tool for evaluating protein energy wasting (PEW) in patients on dialysis. However, the severity of PEW at each stage of chronic kidney disease (CKD), especially with late dialysis initiation, is unclear. **Methods:** We evaluated the MIS of 3659 patients with CKD stages 1–5 and the changes in their MIS results at baseline and at the time before dialysis initiation. Patients were defined to have PEW if they had a subjective global assessment (SGA) rating of C or lower. **Results:** The MIS increased substantially over a follow-up period of 6.12 years for 1124 patients just starting dialysis, with 49.3% having an MIS of 8. The pre-dialysis MIS was associated with baseline MIS, age, cardiovascular disease, and cancer. The prevalence of PEW based on an SGA rating of C or lower increased from 10.5% at baseline to 61.2% immediately before dialysis. The prevalence of PEW based on an MIS of ≥8 increased from 28.5% at baseline to 49.3% immediately before dialysis. In CKD stage 5 patients, 29.4% had PEW based on an MIS of 8 or less, and 11.6% had an SGA rating of C. The MIS was revealed to be associated with renal function, nutritional markers, and cardiometabolic disease (diabetes or cardiovascular disease). **Conclusions:** In conclusion, the MIS increased as CKD progressed to stages 4 and 5, as well as just prior to dialysis. Our study identified patients who required PEW assessment on the basis of their MIS results.

## 1. Introduction

Patients with chronic kidney disease (CKD) experience metabolic and nutritional derangement as the disease progresses; this phenomenon is commonly referred to as protein energy wasting (PEW). Cachexia and loss of muscle fat mass are characteristics of PEW, which causes the loss of somatic and circulating protein and energy reserves [1]. The concept of PEW was introduced by the International Society of Renal Nutrition and Metabolism to describe the concurrent loss of protein and energy stores, with cachexia being the final stage [2]. Patients with advanced-stage CKD or who are on dialysis are at high risk of morbidity and mortality from PEW due to inadequate nutrition intake, inflammation, uremic toxins, acidosis, endocrine disorders, etc., all of which lead to increased breakdown of protein and fat in the body [2]. Furthermore, higher levels of inflammation markers are associated with more rapid CKD progression [3]. Therefore, early detection of PEW is crucial, and early intervention such as nutritional counseling, dietary supplementation, and psychosocial intervention should be considered [4,5].

PEW tends to be more severe in patients with CKD stage 4 or 5 than in those with an earlier CKD stage [6]. Studies have indicated that the prevalence of cachexia or PEW prevalence is less than 2% in patients with CKD stage 1 or 2 [7] and between 11% and 46% in patients with CKD stages 3–5 [8]. In a meta-analysis of 1776 patients with non-dialysis CKD, the prevalence of PEW ranged from 11% to 54% in patients with CKD stages 3–5 [9]. Compared with patients with CKD stage 5 and those on regular dialysis, patients with pre-dialysis CKD and those beginning dialysis may experience more severe PEW because of the accumulation of uremic toxin. According to the *2021 Annual Report on Kidney Disease In Taiwan* [10], the mean average eGFR at the time of dialysis commencement in Taiwan was 5.1 mL/min/1.73 m^2^ in the year 2019. This trend in late dialysis initiation may exacerbate the PEW of patients with pre-dialysis CKD in Taiwan. Several nutrition screening mechanisms are used in clinical practice, but few are specific to CKD, with limited data regarding their validity and reliability [1,11].

Nutritional status abnormalities should be detected and treated early by using practical screening tools to perform thorough nutritional assessments [1,12]. Scoring tools such as the Subjective Global Assessment (SGA) and malnutrition–inflammation score (MIS) are widely used to evaluate the nutritional status of patients on dialysis but are used less frequently in the context of CKD [6,13,14]. The SGA has several advantages, including the speed at which it can be employed, its cost-effectiveness, and simplicity, and it can be completed quickly at the bedside; however, it cannot be used to assess a patient’s visceral protein level because it does not consider biochemical markers [15]. The MIS is a semiquantitative scoring system developed by Kalantar-Zadeh; it combines anthropometric data, biochemical data, and the SGA rating to evaluate PEW in patients on dialysis [1,16,17]. The MIS modified for patients with CKD has 10 components, namely body weight change, dietary intake, gastrointestinal symptoms, functional capacity, comorbidities, fat stores, muscle wasting, body mass index (BMI), albumin level, and total iron-binding capacity (TIBC). Each score component is classified into one of four levels of severity ranging from zero (normal) to three (severely abnormal), and measurements are based on the SGA. Thus, the MIS is more objective than the SGA. Numerous studies have demonstrated that a higher MIS is associated with PEW and with adverse outcomes, particularly with respect to hospitalization and mortality in patients on maintenance hemodialysis [18,19]. Few studies have investigated the MIS in the context of CKD [6,14]. Amparo et al. reported that an MIS of ≥8 was associated with all-cause mortality in 300 patients with CKD stages 3–5 [6]. Furthermore, several biomarkers of PEW were reported to be correlated with decreased eGFR [20,21]; however, various nutritional parameters have exhibited differing patterns of association with the glomerular filtration rate (GFR) [20].

Few studies have thoroughly examined MIS application across all CKD stages. Therefore, longitudinal changes and factors influencing MIS results during the process of CKD diagnosis to the initiation of dialysis should be examined. To obtain a comprehensive understanding of PEW in patients with CKD, we used the SGA and MIS in a large cohort of 3659 patients with CKD stages 1–5, determined which subgroups had a high MIS (MIS ≥ 8) and the factors related to their results, and evaluated the patients’ MIS prior to dialysis and the factors associated with the change in their MIS.

## 2. Materials and Methods

### 2.1. Study Participants

This prospective observational study was conducted in two affiliated hospitals of Kaohsiung Medical University, namely Kaohsiung Medical University Hospital and Kaohsiung Municipal Hsiao-Kang Hospital. Our participants comprised 3749 patients who were enrolled in the Integrated CKD Care Program Kaohsiung for Delaying Dialysis between 11 November 2002, and 31 May 2009. CKD was staged in accordance with the Kidney Disease Outcomes Quality Initiative (KDOQI) guidelines [22], and eGFR was calculated using the four-variable Modification-of-Diet-in-Renal-Disease equation. We excluded patients who were lost to follow-up, had acute kidney injury, experienced an eGFR decrease of more than 50% within 3 months, or had received renal replacement therapy before screening. After patients who were lost to follow-up in less than 3 months were excluded, the final study population comprised 3659 patients with CKD stages 1–5. These patients were followed until 31 December 2014, and informed consent was obtained from all of them. The protocol of this study was approved by the Institutional Review Board of Kaohsiung Medical University Hospital (KMUH-IRB-990198) and was performed in accordance with the ethical standards laid down in the 1964 Declaration of Helsinki.

### 2.2. Data Collection

Baseline demographic characteristics, medical history, and laboratory data were obtained from the participants’ medical records and through interviews with them upon enrollment. MIS and SGA measurements were measured at baseline visit by CKD nurses. We provided the study schedule in supplementary Table S1. Further medical history was obtained through chart reviews conducted by two to three nephrologists. Diabetes mellitus (DM) and hypertension were defined through clinical diagnosis. Hyperuricemia was defined as a uric acid level of >7.2 mg/dL for men and >6.5 mg/dL for women; a participant undergoing urate-lowering therapy was also identified as having hyperuricemia. Cardiovascular disease (CVD) was defined as a clinical diagnosis of ischemic heart disease, congestive heart disease, or cerebrovascular disease. Mean arterial blood pressure and BMI were calculated accordingly. Laboratory data were averaged and analyzed 3 months before and after the implementation of a CKD care system. PEW was defined as having an MIS of ≥8 or an SGA rating of C based on the receiver operating characteristic curve for predicting outcomes in our cohort.

### 2.3. Cardiometabolic Syndrome

Cardiometabolic syndrome is a group of clinical abnormalities that are often associated with DM, cardiovascular disease, and CKD [23]. Several components are considered in the assessment of cardiometabolic syndrome, including central adiposity, triglyceride levels, blood pressure, fasting glucose metrics, and low high-density lipoprotein cholesterol levels [24].

### 2.4. Outcomes

The main study outcome was the MIS assessed immediately before dialysis initiation. The timing of dialysis initiation was based on the regulations established by the National Health Insurance Administration, Ministry of Health and Welfare for initiating dialysis. These regulations specify (i) absolute indications for dialysis (i.e., a GFR of less than 5 mL/min or a serum creatinine level of more than 10 mg/dL) and (ii) relative indications for dialysis (i.e., a GFR of less than 15 mL/min or serum creatinine level of more than 6 mg/dL in addition to the presence of fluid overload or another uremic emergency) [25].

### 2.5. Statistical Analysis

The statistical results pertaining to the baseline characteristics of the participants are expressed as means ± standard deviations for continuous variables with a normal distribution. The significance of the differences between the groups for normally distributed continuous variables was tested using one-way analysis of variance. The significance of the differences between the groups for continuous variables with a nonnormal distribution was tested by conducting Kruskal–Wallis analysis. The intergroup differences in the distributions of categorical variables were tested using the chi-square test. Multivariable linear regression models were used to identify the factors associated with the MIS; these models incorporated adjusted hierarchical covariates, including demographic factors, comorbidity factors, and traditional malnutrition measurements. Statistical analyses were conducted using SPSS 21.0 for Windows (SPSS, Chicago, IL, USA), and a two-sided *p*-value of <0.05 was regarded as statistically significant.

## 3. Results

### 3.1. Characteristics of Patients with CKD Stages 1–5

The baseline demographics, medical history, and clinical and laboratory parameters of the 3659 patients enrolled in this study were stratified by CKD stage and are presented in Table 1. The study cohort had a mean age of 62.3 ± 14.2 years, and 58% of the participants were female. The median eGFR at baseline was 25.2 (12.1–41.8) mL/min/1.73 m^2^, and only 9.7% had CKD stage 1 or 2. Their median urine protein-to-creatinine ratio (UPCR) was 1081 (374–2542), with the highest and lowest ratios being observed in the CKD stage 5 group and CKD stage 3 group, respectively. The percentage of patients with comorbidities was higher in the CKD stage 4 group (Charlson score, 3.7 ± 2.1) and CKD stage 5 group (Charlson score, 3.6 ± 2.1) and lower in the CKD stage 1 + 2 group (Charlson score, 2.4 ± 1.7). The percentages of patients with cardiovascular disease and DM were highest in the CKD stage 5 group and lowest in the CKD stage 3 group. Relative to the CKD stage 3 group, the CKD stage 1 + 2 group had a higher UPCR and higher percentages of patients with cardiovascular diseases and DM. During the follow-up period of 7.47 years, the percentage of patients with end-stage renal disease (ESRD) was highest in the CKD stage 5 group (77.0%); the percentage with all-cause mortality was also highest in this group (34.8%; Table 1).

### 3.2. Malnutrition–Inflammation Across CKD Stages

Serum albumin level and BMI were highest in the CKD stage 3 group and lowest in the CKD stage 5 group, whereas hemoglobin and cholesterol levels decreased from CKD stage 1 + 2 to stage 5 (Table 1). The total MIS (i.e., total score for 10 components) was lowest in the CKD stage 3 group (3.3 ± 2.6) and highest in the CKD stage 5 group (5.7 ± 3.4). The prevalence of PEW (indicating poorer clinical outcomes) as calculated based on an MIS of ≥8 was 7.9% in the CKD stage 3 group and 29.4% in the CKD stage 5 group (Table 1). The prevalence of PEW as calculated based on an SGA rating of C or lower was 3.7% in the CKD stage 3 group and 11.6% in the CKD stage 5 group.

The distributions of the 10 components of the MIS were stratified by CKD stage and are presented in Supplementary Figure S1. The MISs of the patients increased from CKD stage 3 to stage 5 for the 10 associated components (Supplementary Figure S1). Supplementary Figure S1A–J presents the MIS results stratified by CKD stage (i.e., stages 1–5) and MIS component. In patients with advanced CKD, a higher percentage of patients had an MIS of 3 for the components of change in body weight, comorbidities, decreased fat stores, muscle wasting, and serum albumin level.

### 3.3. Factors Associated with Baseline MIS

Table 2 reveals the association of the baseline MIS with the examined baseline factors. Notably, eGFR, DM, cancer, cardiovascular disease, and CRP log were baseline factors that were positively correlated with the MIS, whereas age, hypertension, hemoglobin level, albumin level, cholesterol log, phosphorus level, BMI, and muscle mass index were negatively correlated with the MIS. An MIS of ≥8 was revealed to be more common in patients with cardiometabolic disease than in those without this condition.

We also analyzed the association between baseline SGA results and the examined baseline factors (Supplementary Table S2). Our results indicated that cardiovascular disease and CRP log were positively correlated with MIS results, whereas age, hypertension, hemoglobin level, albumin level, phosphorus level, and BMI were negatively correlated with SGA results. No correlations with eGFR, DM, cancer, cholesterol, or muscle mass index were identified.

### 3.4. Longitudinal Study of Patients Developing ESRD

**Figure 1:**
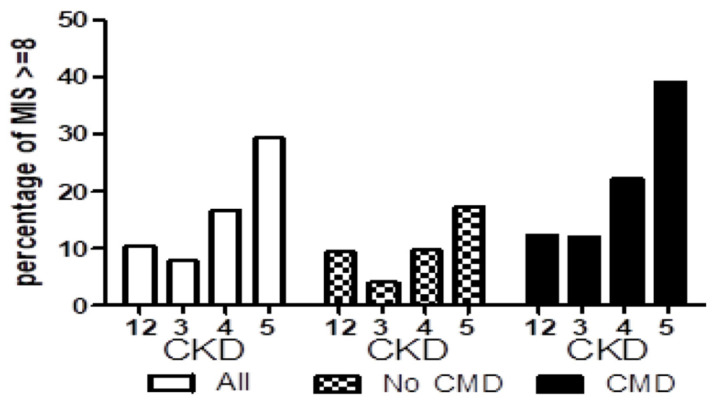
In patients without cardiometabolic disease, the percentage of MIS ≥ 8 was 4.0%, 9.7%, and 17.2% in CKD stages 3, 4, and 5, respectively. In patients with cardiometabolic disease, the percentage of MIS ≥ 8 was 12.0%, 22.2%, and 39.2% in CKD stages 3, 4, and 5, respectively.

**Figure 2:**
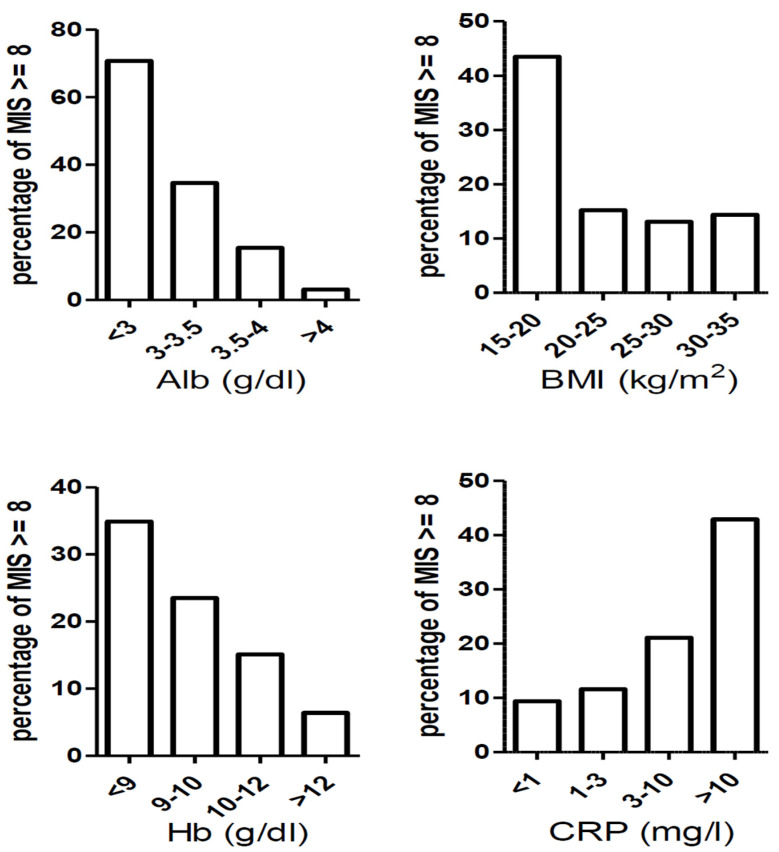
There was a linear trend between the percentage of MIS ≥ 8 and albumin, hemoglobin, and C-reactive protein. There was a higher percentage of MIS ≥ 8 in the group of BMI 15–20 kg/m^2^ compared with other groups.

To study the longitudinal changes in the MIS results, we analyzed the baseline and pre-dialysis data of 1124 patients who developed ESRD during a median follow-up period of 6.12 years (Table 3). The prevalence of PEW based on an MIS of ≥8 increased from 28.5% at baseline to 49.3% immediately before dialysis. The prevalence of PEW based on an SGA rating of C or lower increased from 10.5% at baseline to 61.2% immediately before dialysis. Finally, the scores for nine MIS components (except BMI) increased between the baseline and initiation of dialysis. Figure 1 shows the percentage of MIS ≥ 8 by CKD stages and cardiometabolic disease. Figure 2 reveals the percentage of MIS ≥ 8 by nutritional markers (albumin, BMI, hemoglobin, and CRP). The distributions of the scores for the 10 MIS components from CKD stage 1 to the pre-dialysis stage (5D) are presented in Supplementary Figure S1.

### 3.5. Factors Associated with Pre-Dialysis MIS

Table 4 presents the association of the pre-dialysis MIS with the examined baseline factors. Notably, the pre-dialysis MIS was positively associated with the baseline MIS, age, eGFR, cardiovascular disease, and cancer but negatively associated with CRP log and phosphorus level.

## 4. Discussion

In this cohort study of 3659 patients with CKD stages 1–5, we discovered that PEW was highly prevalent in patients with CKD stage 4 or 5. In the CKD stage 5 group, the prevalence of PEW was 29.4% based on an MIS of ≥8 and 11.6% based on an SGA rating of C or lower. Compared with the SGA, the MIS was associated with more nutritional markers. Furthermore, the MIS was associated with age, eGFR, and cardiometabolic diseases. Over a follow-up period of 6.12 years on average, the pre-dialysis MIS increased substantially, with 49.3% of the patients ultimately having an MIS of ≥8. The pre-dialysis MIS was associated with baseline MIS, age, cardiovascular disease, and cancer. Our study identified the patients who required PEW assessment based on their MIS results. PEW detection and screening should be implemented more proactively for patients with CKD stage 4 because the risk factors associated with PEW play a significant role in the progression of the disease to stage 5.

Patients on dialysis are highly vulnerable to mortality caused by malnutrition [26]. Because the prevalence of malnutrition is high in patients starting dialysis [27], the question of whether malnutrition is a key influencing factor in patients with pre-dialysis CKD is a pertinent topic. Multiple studies have reported various levels of PEW prevalence [7,8,28]. This wide variation in prevalence may be attributable to the differing baseline demographics of the cohorts examined by these studies and the different assessment tools used to evaluate the various aspects of PEW. Consequently, the literature findings are inconclusive with respect to the prevalence of malnutrition in patients beginning specialized pre-dialysis care when malnutrition is measured using a validated method (e.g., the SGA). To assess nutritional deficiencies as a whole, the SGA and MIS can be employed.

The estimated prevalence of CKD in Taiwan was reported to be approximately 22.6% per million population [29]. In 2019, the incidence of ESRD was increasing, with diabetic nephropathy accounting for 48.6% of new ESRD cases [10]. In Taiwan, dialysis tends to be started late, with the Taiwan Renal Registry reporting an eGFR of 5.1 mL/min/1.73 m^2^ for the year 2019 [10]. With late dialysis initiation, patients with CKD have longer periods of malnutrition and inflammation, which promote earlier mortality. This treatment trend is reflected in our study, in which malnutrition and inflammation factors were prominent at baseline and immediately before dialysis initiation. In such circumstances, the SGA, which is based on multiple subjective components, may be less sensitive in assessing malnutrition during the early stages of CKD. The effectiveness and accuracy of nutritional screening can be improved through objective data assessment methods, such as anthropometric measurements, when patients are at an earlier stage of CKD. In our cohort, BMI scores (MIS 8) increased considerably after CKD stage 4, and this trend was stronger in patients with cardiometabolic disease (CMD) (Figure 1). Accordingly, early PEW detection and screening should be implemented more proactively for patients with CKD stage 4 because the risk factors associated with PEW play a key role in the transition of the disease to stage 5. A key component of controlling the progression of CKD is the strategy of nutrition intervention. Lower dietary protein intake has been shown to slow CKD progression and improve albuminuria for decades. Recently, there has been evidence that a plant-based, fiber-rich, low-protein diet may alter the gut microbiome, and nutraceutical products that contain isoflavones may reduce cardiovascular risk while modulating uremic toxin production and slowing CKD progression [30,31].

The MIS is highly applicable in clinical practice. For patients on hemodialysis, the MIS has played a major role in predicting mortality [32,33]. According to Vogt et al., the MIS is a significant predictor of 2-year mortality in patients on hemodialysis [33]. In a 5-year prospective cohort study conducted by Rambod et al., every 2-unit increase in the MIS was associated with a 2-fold increase in the risk of death of patients on hemodialysis with an MIS of ≥5 [19]. However, few studies have examined the effects of MIS on CKD progression. In patients with CKD, malnutrition is linked to higher mortality and hospitalization rates [33]. Dietary restriction and insufficient food intake related to poor appetite can lead to malnutrition; these factors, coupled with various comorbid conditions, accelerate CKD progression. Furthermore, our previous study indicated that low blood pressure contributes to the detrimental cycle of malnutrition and inflammation in CKD, resulting in poor outcomes [34]. The research on the prevalence of PEW and potential risk factors for PEW in patients with pre-dialysis CKD is limited. To the best of our knowledge, the cohort examined in this study is the largest to date in which the MIS has been evaluated in patients with pre-dialysis CKD. The pre-dialysis MIS was revealed to be associated with the baseline MIS, age, eGFR, cardiovascular disease, and cancer. Our data indicated that specific groups (e.g., older patients with CMD) should be prioritized for early intervention in the form of multidisciplinary programs and dietetic interventions for combating PEW.

On the basis of the 2020 update of the KDOQI Clinical Practice Guidelines for Nutrition in CKD, use of the MIS is recommended for patients on maintenance hemodialysis or those who have undergone post-transplantation; however, the guidelines do not include recommended cutoff levels [1]. An MIS of <10 has been reported to suggest adverse outcomes in patients with CKD. In a study conducted in Brazil, the MIF cutoff value of 7 was identified as a predictor of mortality in patients on hemodialysis at various follow-up points [35]. Jagadeswaran et al. examined a CKD cohort and discovered that an MIS of ≥7 indicated a high risk of mortality in patients with pre-dialysis CKD [14]. In this study, an MIS cutoff of 8 predicted the CKD progression of a CKD cohort.

The prevalence of CKD in older adults is high, being up to 30% [36]. Aging is widely recognized as a main risk factor for chronic disease development. Notably, older adults are more prone to disease-related weight loss, sarcopenia, and frailty syndrome, all of which negatively affect clinical outcomes [37,38]. In a study of a random sample of Italians, De Nicola et al. discovered that CKD is associated with age, obesity, hypertension, DM, and background cardiovascular disease [39]. Several studies have demonstrated that CKD is a strong risk factor for cardiovascular disease, especially in patients with acute coronary syndrome [40,41]. The development of atherosclerosis caused by inflammation and malnutrition, both of which have been identified as nontraditional risk factors for CKD, can lead to CV events or mortality [42]. Our data revealed that age and cardiometabolic diseases are associated with the MIS of patients with pre-dialysis CKD. Thus, patients with pre-dialysis CKD should be prioritized for early intervention in the form of CKD programs.

The main strength of this study is its large sample comprising CKD patients. This is the first study to analyze MIS results in a large population of pre-dialysis patients. Nevertheless, there are several limitations. First, we adopted a retrospective design, and MIS measurements did not necessarily indicate a steady status. Physicians may decide to obtain MIS measurements simply when they suspect malnutrition or inflammation. Second, the MIS measurements for each CKD stage and before dialysis were not obtained at consistent intervals, preventing us from accurately estimating changes. Third, this study examined an East Asian cohort that commenced dialysis late, making it impossible to examine the impact of ethnicity. Fourth, we did not consider the dietary and medication factors that might have had influence on the outcomes of the patients in our study. A diet that restricts protein can result in malnutrition, while incorporating ESA and ketoanalogues of amino acid (Ketosteril^®^) can lead to an improvement in nutrition status. Fifth, patients were enrolled from a single center, and the balance between taking a low-protein diet and ensuring adequate nutrition intake in CKD education could have differed between this center and other centers. Sixth, in this study, the effects of increasing exercise and physical activity on both MIS and PEW were not evaluated. Lastly, we did not measure interobserver or intra-observer errors; however, the distribution between two affiliated hospitals was similar. Thus, a large and diverse sample is required to confirm the findings of our study.

## 5. Conclusions

In conclusion, we demonstrated that PEW is highly prevalent in patients with CKD stages 4 and 5. The MIS is a useful tool for identifying PEW. Notably, the MIS increased gradually from CKD stages 1 to 3 and dramatically from CKD stage 4 to immediately before dialysis. In addition to nutritional markers, the MIS was associated with age and cardiometabolic disease. To guide the treatment and improve the outcomes of pre-dialysis patients, clinicians should identify high-risk patients on the basis of modifiable clinical factors such as the MIS.

## Figures and Tables

**Table 1:** Baseline characteristics of CKD stage 1–5 patients.

Variable	CKD Stage				*p* Value
	1 + 2	3	4	5	
**No. of patients**	356 (9.7%)	1183 (32.3%)	961 (26.3%)	1159 (31.7%)	<0.001
Age (year)	51.8 (15.8)	63.5 (13.3)	65.0 (13.8)	62.1 (13.4)	<0.001
Sex (female)	143 (40.2%)	309 (26.1%)	447 (46.5%)	638 (55.0%)	<0.001
**Comorbidity**					
Cardiovascular disease	29 (8.1%)	256 (21.6%)	268 (27.9%)	337 (29.1%)	<0.001
Diabetes mellitus	148 (41.6%)	558 (47.2%)	524 (54.5%)	578 (49.9%)	<0.001
Hypertension	179 (50.3%)	695 (58.7%)	639 (66.5%)	850 (73.3%)	<0.001
Severe liver disease	16 (4.5%)	55 (4.6%)	51 (5.3%)	56 (4.8%)	0.888
Cancer	20 (5.6%)	79 (6.7%)	91 (9.5%)	123 (10.6%)	<0.001
Charlson score	2.4 (1.7)	3.3 (2.0)	3.7 (2.1)	3.6 (2.1)	<0.001
Mean BP (mmHg)	99.0 (13.1)	99.3 (13.2)	99.6 (13.9)	100.9 (14.2)	0.017
**Laboratory data**					
eGFR (mL/min/1.73 m^2^)	77.8 (68.0–95.8)	41.1 (35.2–47.8)	22.2 (18.4–26.0)	8.9 (6.5–11.7)	<0.001
UPCR (mg/g)	660(147–2547)	397(69–1071)	1218(540–2731)	1903(1088–4035)	<0.001
Hba1c (%)	6.5 (1.6)	6.8 (2.0)	6.6 (1.6)	6.7 (1.8)	<0.001
WBC (×1000/uL)	7.2 (2.3)	7.1 (2.4)	7.1 (2.2)	7.4 (2.3)	0.008
Hemoglobin (g/dL)	13.8 (2.0)	12.8 (2.0)	10.8 (1.8)	9.1 (1.4)	<0.001
Total cholesterol (mg/d)	205 (173–239)	192 (165–221)	192 (165–225)	188 (156–217)	<0.001
Triglyceride (mg/dL)	127 (82–189)	124 (89–186)	132 (98–194)	123 (87–176)	<0.001
Phosphorus (mg/dL)	3.7 (0.8)	3.7 (0.7)	4.2 (0.9)	5.4 (1.4)	<0.001
Calcium (mg/dL)	9.3 (0.6)	9.3 (0.6)	9.1 (0.7)	8.8 (0.9)	<0.001
**Malnutrition–inflammation**					
Albumin (g/dL)	3.9 (0.7)	4.0 (0.5)	3.8 (0.5)	3.7 (0.5)	<0.001
Body mass index (kg/m^2^)	24.8 (4.0)	25.4 (4.5)	25.1 (3.7)	25.0 (4.2)	<0.001
Muscle mass index(kg/m^2^)	3.3 (0.5)	3.4 (0.6)	3.4 (0.4)	3.3 (0.5)	0.005
C-reactive protein (mg/L)	0.8 (0.2–4.9)	0.9 (0.3–3.6)	1.3 (0.5–5.5)	1.5 (0.5–7.0)	<0.001
MIS	3.7 (2.8)	3.3 (2.6)	4.5 (3.1)	5.7 (3.4)	<0.001
MIS ≥ 8	37 (10.4%)	94 (7.9%)	160 (16.6%)	341 (29.4%)	<0.001
SGA C	16 (4.5%)	44 (3.7%)	59 (6.1%)	134 (11.6%)	<0.001
**Outcomes**					
ESRD	19 (5.3%)	137 (11.6%)	391 (40.7%)	893 (77.0%)	<0.001
All-cause mortality	20 (5.6%)	199 (16.8%)	305 (31.7%)	403 (34.8%)	<0.001

Abbreviations: BP: blood pressure; eGFR: estimated glomerular filtration rate; UPCR: urine protein-to-creatinine ratio; WBC: white blood count; ESRD: end-stage renal disease; MIS: malnutrition–inflammation score; SGA: subjective global assessment. Data are presented as mean (standard error), median (interquartile range), or count (percentage %).

**Table 2:** Association of baseline MIS and baseline factors.

	MIS	
Variables	Beta Coefficient (95% CI)	*p*
Age (years)	−0.010 (−0.016 to −0.004)	<0.001
Gender (male)	0.114 (−0.061 to 0.290)	0.202
eGFR (ml/min/1.73 m^2^)	0.006 (0.002 to 0.010)	0.001
UPCR log	0.064 (−0.092 to 0.221)	0.421
Diabetes	0.283 (0.113 to 0.454)	<0.001
Cardiovascular disease	1.132 (0.920 to 1.345)	<0.001
Cancers	0.644 (0.349 to 0.939)	<0.001
Smokers	−0.062 (−0.290 to 0.167)	0.596
Hypertension	−0.266 (−0.429 to −0.102)	0.001
Hemoglobin (g/dL)	−0.110 (−0.156 to −0.064)	<0.001
Cholesterol log	−1.350 (−2.069 to −0.632)	<0.001
Mean BP (mmHg)	−0.007 (−0.013 to 0.002)	0.011
Body mass index (kg/m^2^)	−0.102 (−0.121 to −0.082)	<0.001
Muscle mass index (kg/m^2^)	−0.240 (−0.778 to −0.023)	0.025
Albumin(g/dL)	−2.810 (−2.980 to −2.640)	<0.001
CRP log	0.924 (0.840 to 1.009)	<0.001
Phosphorus (mg/dL)	−0.155 (−0.256 to −0.054)	0.003

Abbreviations: MIS: malnutrition–inflammation score; eGFR: estimated glomerular filtration rate; UPCR: urine protein-to-creatinine; CRP: C-reactive protein.

**Table 3:** Baseline and pre-dialysis MIS in patients who underwent dialysis.

Variable	Baseline	Pre-Dialysis	*p* Value
Age (year)	60.8 (13.6)	66.5 (13.6)	-
Sex (female)	559 (49.4%)	559 (49.4%)	-
Charlson score	3.4 (2.1)	3.6 (2.2)	0.125
eGFR (ml/min/1.73 m^2^)	30.6 (24.3)	4.9 (2.8)	<0.001
**Malnutrition–inflammation**			
MIS	5.7 (3.2)	7.8 (3.2)	<0.001
MIS ≥ 8	320 (28.5%)	554 (49.3%)	<0.001
SGA C	118 (10.5%)	688 (61.2%)	<0.001
MIS1 (change in body weight)	108 (9.5%)	513 (45.6%)	<0.001
MIS2 (dietary intake)	264 (23.3%)	649 (57.8%)	<0.001
MIS3 (GI symptoms)	250 (22.1%)	935 (82.6%)	<0.001
MIS4 (functional capacity)	234 (20.7%)	648 (57.7%)	<0.001
MIS5 (comorbidities)	482 (42.6%)	601 (53.5%)	0.015
MIS6 (decreased fat stores)	165 (14.6%)	239 (21.3%)	0.016
MIS7 (muscle wasting)	238 (21.0%)	367 (32.7%)	<0.001
MIS8 (body mass index)	126 (11.1%)	182 (16.2%)	0.135
MIS9 (serum albumin)	814 (71.9%)	955 (85.0%)	<0.001
MIS10 (serum TIBC)	540 (48.0%)	540 (48.0%)	-

Abbreviations: eGFR: estimated glomerular filtration rate; MIS: malnutrition–inflammation score; SGA: subjective global assessment; GI: gastrointestinal; TIBC: total iron binding capacity. Percentage of score > 0 in each MIS component.

**Table 4:** Association of the pre-dialysis MIS with baseline factors.

	Pre-Dialysis MIS	
Variables	95% CI Beta Coefficient	*p*
Baseline MIS	0.751 (0.692 to 0.810)	<0.001
Follow up years	−0.000 (0.000 to 0.000)	0.872
Age (years)	0.014 (0.004 to 0.024)	0.007
Gender (female)	0.240 (−0.040 to 0.521)	0.093
eGFR (ml/min/1.73 m^2^)	0.019 (0.001 to 0.036)	0.038
UPCR log	0.134 (−0.248 to 0.515)	0.492
Diabetes	−0.158 (−0.478 to 0.162)	0.332
Cardiovascular disease	0.672 (0.334 to 1.010)	<0.001
Cancer	0.594 (0.142 to 1.047)	0.010
Smoker	−0.003 (−0.453 to 0.446)	0.988
Hypertension	0.072 (−0.230 to 0.375)	0.640
Hemoglobin (g/dL)	0.038 (−0.054 to 0.130)	0.416
Cholesterol log	−0.285 (−1.456 to 0.885)	0.632
Mean BP (mmHg)	−0.007 (−0.017 to 0.003)	0.160
Body mass index (kg/m^2^)	−0.029 (−0.063 to 0.006)	0.105
Muscle mass index(kg/m^2^)	−0.041 (−0.174 to 0.092)	0.552
Albumin (g/dL)	0.207 (−0.133 to 0.547)	0.232
CRP log	−0.177 (−0.338 to −0.016)	0.031
Phosphorus (mg/dL)	−0.113 (−0.218 to −0.008)	0.034

Abbreviations: MIS: malnutrition–inflammation score; eGFR: estimated glomerular filtration rate; UPCR: urine protein-to-creatinine; BP: blood pressure; CRP: C-reactive protein.

## Data Availability

The data used in this study are available from the corresponding author upon reasonable request. The data are not publicly available due to privacy.

## Supplementary Materials

The following supporting information can be downloaded at: https://www.mdpi.com/article/10.3390/nu16234014/s1, Table S1: Association of baseline SGA with nutrition markers; Table S2: Study schedule of laboratory measurements and procedures; Figure S1: The distributions of the scores for the 10 MIS components from CKD stage 1 to the pre-dialysis stage (5D).
