# Supplementary material for: Malnutrition–Inflammation Score of Patients with Chronic Kidney Disease from Early Stage to Initiation of Dialysis
Source: Nutrients. 2024 Nov 23;16(23):4014. doi: 10.3390/nu16234014 (PMC11643849; doi:10.3390/nu16234014)
Supplement: Supplementary file 1 [file nutrients-16-04014-s001.zip › Supplement Table S1_new.pdf]

Supplementary Table S1. Study schedule of laboratory measurements and procedures

Parameters	Screen	Baseline	3rd mo	6th mo	9th mo	1st yr	Pre-dialysis
Informed consent	●						
Screen labs	●						
Demographic information	●						
Eligibility confirmation	●						
Medical history		●				●	
Drug history		●				●	
CKD education		●	○	○	○	○	
CKD achievement evaluation						○	
Nutrition evaluation and education		●		○		○	
Predialysis evaluation and education						○	●
Anthropometric measures		●		○		○	
CBC		●	○	○	○	●	●
eGFR		●	○	○	○	●	●
UPCR		●	○	○	○	●	●
UACR		●				●	●
Urinary analysis		●		○		●	●
Nutritional profile		●		○		●	●
Lipid profile		●				●	●

**Electrolyte profile**		●		○		●	●
**Diabetic profile**		●		○		●	●
**Mineral profile**		○		○		○	●
**Iron profile**		○				○	
**Hepatitis profile**		○					
**Electrocardiogram**		○				○	
**Echocardiogram**		○				○	
**Chest X ray**		○				○	
**Bioelectric impedance**						◇	
**Ankle brachial index**						◇	
**Pulse wave velocity**						◇	
**KDQOL quality of life**						◇	
**Diet History Questionnaire**						◇	
**Mini Mental Status Exam**						◇	
**Beck Depression Inventory**						◇	
**Physical Activity**						◇	
**Genetic blood sample**						◇	

Abbreviations: CBC: complete blood count; eGFR: estimated glomerular filtration rate; UPCR:urine protein-creatinine ratio; UACR:urine albumin-creatinine ratio.

● Both integrated care program and regular care program
○ Only integrated care program
◇ Randomly selected patients in the integrated care program
