# Supplementary material for: Malnutrition–Inflammation Score of Patients with Chronic Kidney Disease from Early Stage to Initiation of Dialysis
Source: Nutrients. 2024 Nov 23;16(23):4014. doi: 10.3390/nu16234014 (PMC11643849; doi:10.3390/nu16234014)
Supplement: Supplementary file 1 [file nutrients-16-04014-s001.zip › Supplement Table S2_new.pdf]

Supplement Table S2.

Association of baseline SGA with nutrition markers

	SGA	
Variables	95% CI Beta coefficient	***P***
Age (years)	-0.007 (-0.010 to -0.004)	<0.001
Gender (female)	0.070 (-0.033 to 0.173)	0.183
eGFR (ml/min/1.73 m^2)	0.002 (-0.001 to 0.004)	0.171
UPCR log	-0.043 (-0.135 to 0.048)	0.353
Diabetes	0.086 (-0.013 to 0.186)	0.090
Cardiovascular disease	0.161 (0.037 to 0.285)	0.011
Cancer	-0.029 (-0.202 to 0.143)	0.739
Smoker	-0.063 (-0.197 to 0.071)	0.354
Hypertension	-0.186 (-0.282 to -0.091)	<0.001
Hemoglobin (g/dl)	-0.061 (-0.088 to -0.035)	<0.001
Cholesterol log	-0.412 (-0.833 to 0.009)	0.055
Mean BP (mmHg)	-0.000 (-0.0004 to 0.003)	0.792
Body mass index (kg/m^2)	-0.034 (-0.045 to -0.022)	<0.001
Muscle mass index(kg/m^2)	-0.080 (-0.239 to 0.079)	0.324
Albumin (g/dl)	-0.531 (-0.630 to -0.431)	<0.001
CRP log	0.428 (0.379 to 0.478)	<0.001
Phosphorus (mg/dl)	-0.103 (-0.163 to -0.044)	0.001

Abbreviations: MIS: malnutrition-inflammation score; eGFR: estimated glomerular filtration rate; UPCR: urine protein-to-creatinine; BP: blood pressure; CRP: C-reactive protein; TIBC: total iron binding capacity.
