# Supplementary figures and images for: Malnutrition–Inflammation Score of Patients with Chronic Kidney Disease from Early Stage to Initiation of Dialysis
Source: Nutrients. 2024 Nov 23;16(23):4014. doi: 10.3390/nu16234014 (PMC11643849; doi:10.3390/nu16234014)

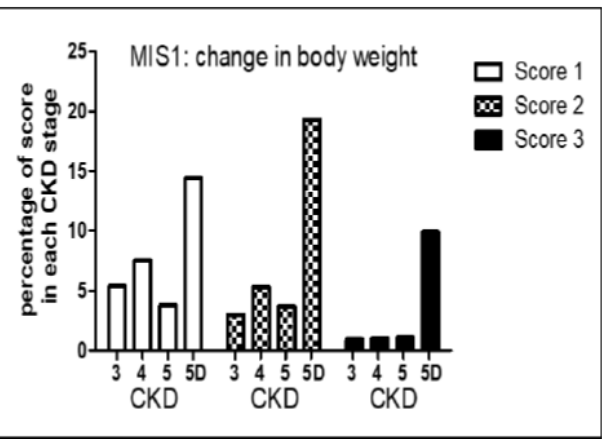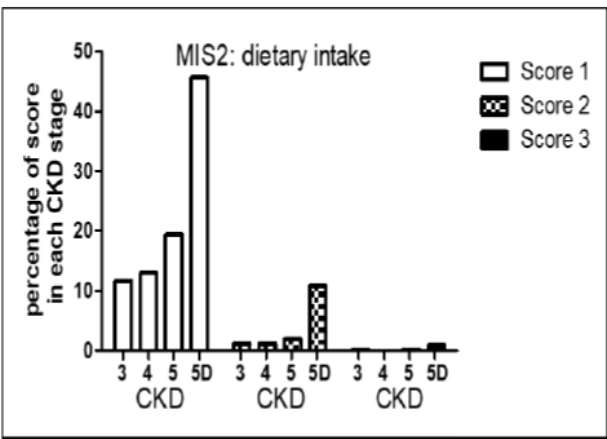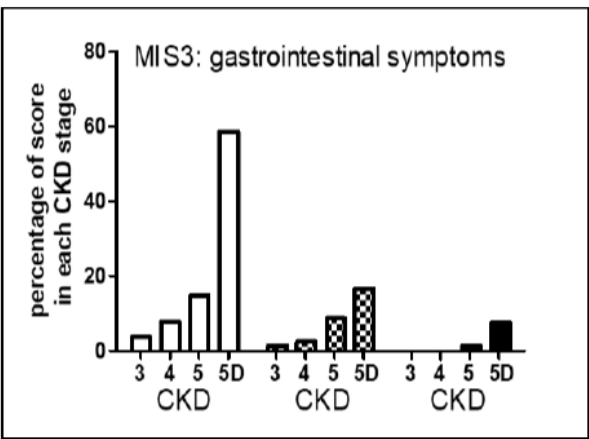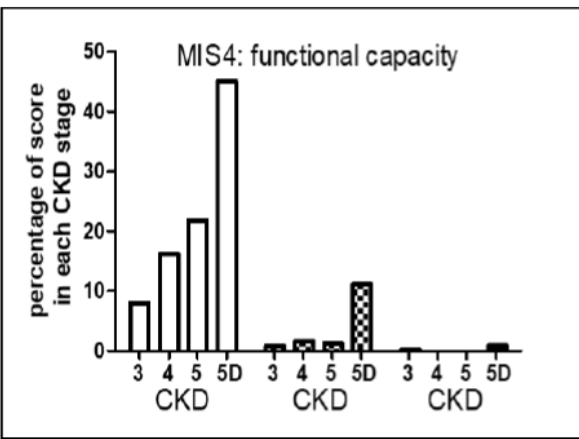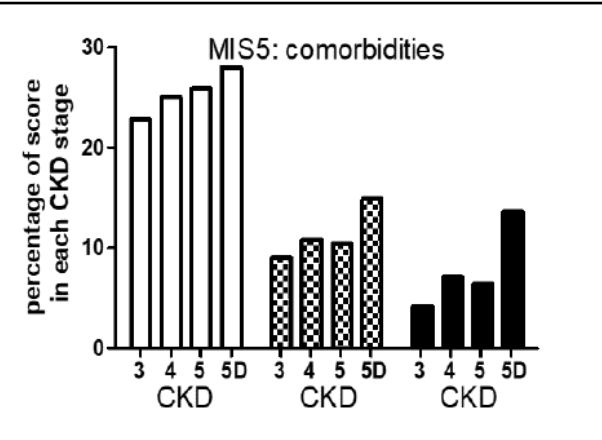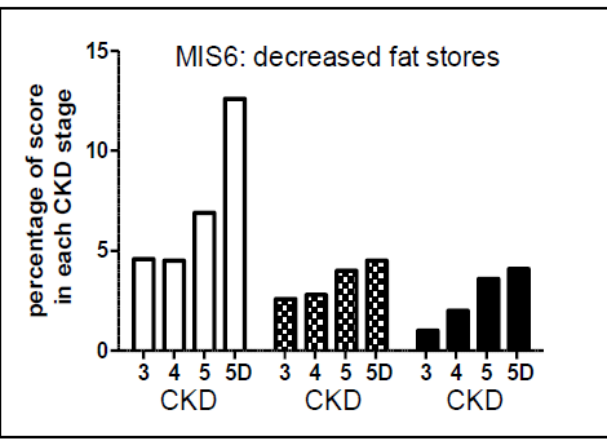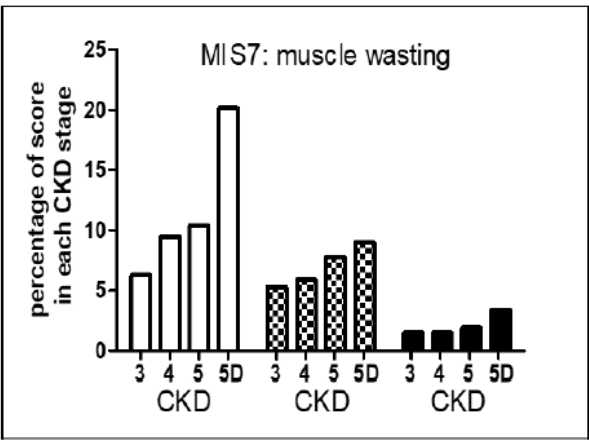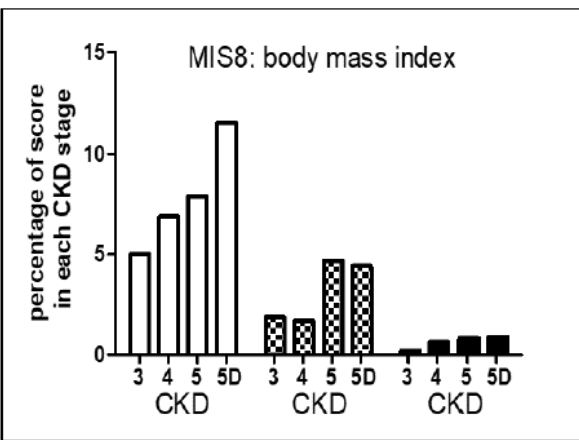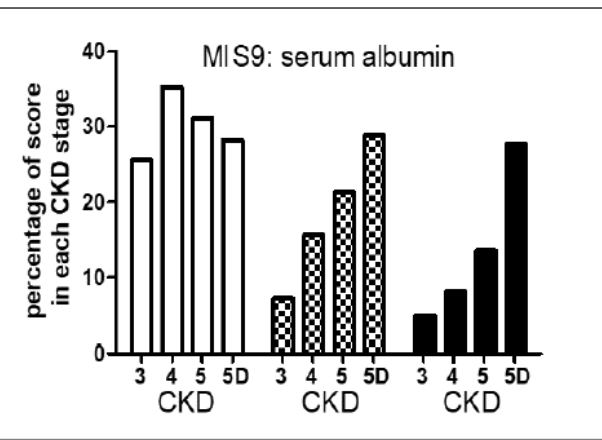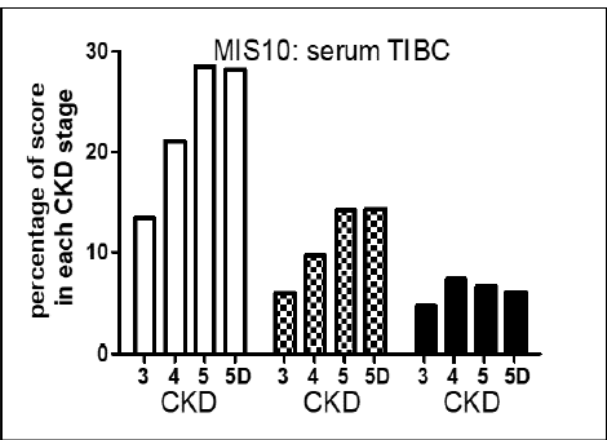

**Supplementary Figure S1**

Supplement: Supplementary file 1 [file nutrients-16-04014-s001.zip › Supplementary Figure S1_new.pdf]
